# Difficultés de prise en charge de la maladie de Basedow chez l’enfant: à propos d’un cas

**DOI:** 10.11604/pamj.2018.30.183.15325

**Published:** 2018-06-27

**Authors:** Nassiba Elouarradi, Ghizlane El Mghari, Nawal El Ansari

**Affiliations:** 1 Service d’Endocrinologie, Diabétologie et Maladies Métaboliques, CHU Mohamed VI, Faculté de Médecine et de Pharmacie, Université Cadi Ayyad, Marrakech, Maroc

**Keywords:** Basedow, enfant, traitement, Graves, child, treatment

## Abstract

La prise en charge adéquate de la maladie de Basedow chez l'enfant reste un sujet de controverse en endocrinologie pédiatrique et la durée optimale du traitement médical pour induire une rémission de la maladie ainsi que les indications des thérapeutiques alternatives, restent à définir. Nous rapportons l'observation d'une enfant âgée de 11 ans, sans antécédents pathologiques particuliers, qui consulte pour un amaigrissement progressif associé à des diarrhées. C'est devant la survenue d'une tuméfaction cervicale antérieure constatée par l'entourage que les parents décident de consulter. Les données cliniques et paracliniques ont permis de retenir le diagnostic de la maladie de Basedow. La patiente a été mise sous traitement médical : Carbimazole. L'évolution a été marquée par l'apparition d'une thrombopénie suggérant une administration prudente du traitement médical et devant l'absence de rémission après 4 ans de traitement, une irathérapie a été indiquée. Bien que rare, la maladie de Basedow reste la première cause de l'hyperthyroïdie chez l'enfant. Son diagnostic positif est facile mais sa prise en charge peut poser d'énormes problèmes. Le traitement médical se base sur les antithyroïdiens de synthèse mais qui n'est pas toujours anodin comme pour le cas de notre patiente .la thyroïdectomie subtotale ou le traitement par iode radioactif sont les deux alternatives thérapeutiques. De diagnostic positif facile, la maladie de Basedow est une maladie rare et sévère chez l'enfant et qui pose surtout des difficultés de prise en charge.

## Abstract (en)

Adequate management of Graves’ disease in children is an area of controversy in pediatric endocrinology, while optimal duration of medical treatment inducing remission in disease as well as indications for therapeutic alternatives still needs to be determined. We report the case of a 11-year old female child with no particular past medical history., presenting with progressive weight loss associated with diarrhea. Her parents brought the child to hospital because neighbours noticed anterior cervical swelling on her. Clinical and paraclinical data helped to retain the diagnosis of Graves’ disease. The patient underwent medical treatment with carbimazole. Patient’s evolution was marked by the occurrence of thrombocytopenia, suggesting prudent administration of medical treatment. Iratherapy was indicated due to the absence of remission after 4 years of treatment. Although rare, Graves’ disease is the first cause of hyperthyroidism in children. Positive diagnosis is easy but its management may pose enormous problems. Medical treatment is based on synthetic antithyroid drugs, but they are not always innocuous, as in the case of our patient. Then subtotal thyroidectomy or radioactive iodine treatment are two therapeutic alternatives. Graves’ disease is a rare and severe disease in children posing considerable management difficulties.

## Introduction

La maladie de Basedow est une maladie rare et sévère, qui touche surtout le grand enfant avec prédominance féminine nette. L'hyperthyroïdie en raison de la production d'anticorps anti - récepteur de la thyréostimuline (TSH) qui stimulent la thyroïde induisant la production excessive d'hormones thyroïdiennes. Le diagnostic est posé devant un tableau clinique très évocateur regroupant le plus souvent un goitre, une exophtalmie et un tableau de thyrotoxicose, confirmé sur le plan biologique par présence d'une hyperthyroïdie avec des anticorps anti-récepteurs TSH positifs et sur le plan radiologique par un goitre diffus, homogène, hypervasculaire et anodulaire. La régression rapide et spontanée de la maladie de Basedow est rare. En l'absence de consensus actuel sur le traitement de choix, les approches thérapeutiques de l'hyperthyroïdie restent les antithyroïdiens de synthèse, l'iode radioactif ou la chirurgie. Nous rapportons l'observation d'une enfant chez qui la maladie de Basedow a posé d'énormes difficultés de prise en charge.

## Patient et observation

Il s'agit d'une enfant âgée de 11 ans, issue d'un mariage non consanguin, 3^ème^ d'une fratrie de 4, scolarisée, sans antécédents de thyréopathie familiale ou d'autres atteintes auto-immunes, qui consulte pour un amaigrissement progressif associé à des diarrhées liquidiennes évoluant depuis l'âge de 7 ans. C´est devant la survenue d´une tuméfaction cervicale antérieure constatée par l´entourage que les parents décident de consulter. L'examen clinique a objectivé une tachycardie à 102 battements /minute avec une exophtalmie bilatérale avec à l'examen cervical un goitre diffus homogène et un souffle à l'auscultation. Au bilan, l'hyperthyroïdie biologique a été confirmée avec une TSH freinée à 0,056 Uui/l, Le bilan immunologique a objectivé des anticorps anti-récepteur TSH positifs à 14,46 UI/ml et des anti-TPO positifs à 287,815 UI/ml. Sur le plan radiologique, une échographie cervicale a été faite objectivant un goitre diffus homogène. La patiente a été mise sous Carbimazole avec une dose initiale de 10 mg/jour associé à un B-bloquant. La NFS de contrôle a objectivé l'apparition d'une thrombopénie isolée à 57000/mm^3^. La dose de Carbimazole a été diminuée à 7,5 mg/jours et la NFS de contrôle à objectivé un taux de plaquettes à 88000/mm^3^. L'évolution était marquée, après 2 ans de traitement, sous schéma combiné associant carbimazole et levothyroxine par une normalisation biologique, avec persistance du goitre. A 4 ans de la première poussée, nous avons noté une rechute avec réapparition d'une hyperthyroïdie à 38,11 pmol/l de T4. La dose de Carbimazole a été ré augmentée à 10 mg/j. Devant la découverte d'une thrombopénie à 57000/mm^3^ à la NFS de contrôle avec l'apparition d'ecchymoses cutanées la patiente a été adressée pour irathérapie.

## Discussion

La maladie de Basedow est rare chez l'enfant. Elle survient chez 0,02 % des enfants (1/5000) soit 1-5 % des patients avec une maladie de Basedow [1]. Le diagnostic positif de cette maladie est souvent facile mais la prise en charge adéquate de cette maladie reste un sujet de controverse en endocrinologie pédiatrique et la durée optimale du traitement médical pour induire une rémission de la maladie, reste à définir [2,3]. Le traitement médical par les anti-thyroidiens de synthèse est toujours tenté en première intention. L'utilisation de B-bloquants doit être recommandée à la phase initiale de la maladie afin de faire disparaitre les symptômes adrénergiques. Les antithyroïdiens de synthèse les plus fréquemment utilisés sont les thionamides: Carbimazole (Neomercazole^®^) et son métabolite actif, Methimazole (Tapazole^®^). Ces médicaments inhibent la synthèse des hormones thyroïdiennes en interférant avec l'iodation des résidus tyrosines par la thyroperoxydase dans la thyroglobuline. La posologie initiale du Carbimazole ou du Methimazole est de 0,5 à 0,8 mg/kg/jour et au maximum de 30 mg/jour. Le Propylthiouracile (PTU) permet aussi de bloquer la conversion de T4en T3 [4]. Tous les thionamides sont associés à des réactions indésirables mineures (éruption cutanée, urticaire, arthralgies, troubles gastro-intestinaux) dans environ 5- 25 % des cas. Le principal effet indésirable grave des anti-thyroidiens de synthèse est l'agranulocytose avec une fréquence observée entre 0,2 et 0,5% des cas pour tous les médicaments de cette classe. Chez notre malade l'effet indésirable constaté est la thrombopénie isolée. La fréquence des effets indésirables dose dépendants et celle des effets indésirables graves est très faible chez les patients recevant du Carbimazole ou Methimazoleà une dose inférieure à 10 mg par jour [5]. Pour la surveillance du traitement médical, on préconise un dosage de la T4 libre (ou de la T3 Libre en cas d'hyperthyroïdie à T3) à la 4ème semaine. Une fois l'euthyroidie obtenue on préconise un dosage de la T4 libre et de la TSH tous les 3 à 4 mois. Une surveillance de la NFS tous les 10 jours doit se faire pendant les 2 premiers mois et avertir le patient d'arrêter le traitement et de faire une NFS en cas de fièvre [6]. Une fois normalisation es concentrations plasmatiques de T4 libre et T3libre obtenue, deux méthodes thérapeutiques sont possibles: 1) diminuer les doses du Carbimazole afin de retrouver ou de maintenir l'euthyroidie; 2) continuer à utiliser des doses induisant une insuffisance thyroïdienne modérée qui sera compensée par une dose efficace de L-thyroxine.

En cas de rechute, une seconde cure peut être envisagée, sinon, la thyroïdectomie sera le plus souvent la solution proposée aux patients. Néanmoins, comme pour les adultes, les facteurs qui sont décrits comme prédictifs de la rechute de la maladie de Basedow sont: l'âge, la taille du goitre, la sévérité initiale de la maladie, le délai nécessaire à la survenue de l'euthyroidie, les concentrations des anticorps anti-RTSH au diagnostic et à la fin du traitement ainsi que la durée du traitement médical initial [7-9]. Pour la chirurgie, la thyroïdectomie totale est souvent préférée à la thyroïdectomie partielle (ou subtotale) afin de réduire le risque l'hyperthyroïdie récurrente. La vascularisation de la glande peut être réduite en associant au traitement par les ATS, un traitement par l'iode (5-10 gouttes de solution Lugol), pendant la semaine qui précède la chirurgie. Le traitement substitutif par L-thyroxine doit être débuté dans les jours qui suivent le geste chirurgical [4]. Le traitement par iode radioactif est efficace chez les enfants qui présentent une hyperthyroïdie due à la maladie de BASEDOW [10]. Il est réservé aux rechutes après traitement chirurgical. Cette attitude n'est peut-être pas justifiée, et les résultats des études déjà publiées devraient nous pousser à mettre en route des protocoles thérapeutiques incluant l'iode radioactif La destruction de la glande thyroïde induite par l'iode radioactif est obtenue dans la majorité des cas par une seule dose orale. La thérapie radioactive a également un effet sur l'auto -immunité thyroïdienne. Des doses plus importantes (220-275uCi/g équivalents à environ 250 Gy) doivent être préférées aux doses plus faibles. Le traitement radioactif comporte un risque modéré d'aggravation des signes oculaires de la maladie. Il n'y a actuellement aucune preuve d'un retentissement sur la fonction de reproduction des patients ayant été traités pendant la période pédiatrique, ni d'une augmentation de la fréquence de malformations congénitales au sein de la descendance [11]. En raison de craintes sur les risques à long terme potentiellement liés à ce traitement, des essais randomisés contrôlés restent nécessaires pour répondre définitivement à ces questions. Dans tous les cas, le risque d'hypothyroidie définitive après traitement est élevé et nécessite une hormonothérapie de substitution à vie par L-thyroxine et une surveillance appropriée [4]. Chez notre patiente, une irathérapie a été proposée devant la présence d'une contre-indication à la majoration du traitement médical et à la chirurgie. Le suivi prolongé jusqu'à l'âge adulte est nécessaire, même après la fin de l'administration du traitement médical ou après un traitement radical afin de déterminer l'efficacité de la prise en charge des patients pendant l'enfance et l'impact sur leur santé générale.

## Conclusion

Certes, le diagnostic positif de la maladie de Basedow chez l'enfant est souvent facile mais la prise en charge adéquate de cette maladie reste un sujet de controverse en endocrinologie pédiatrique. Le traitement médical par les antithyroïdiens de synthèse est toujours tenté en première intention. Les autres alternatives thérapeutiques sont la chirurgie et la radiothérapie.

## Conflits d’intérêts

Les auteurs ne déclarent aucun conflit d'intérêts.
